# Unraveling the discrepancies between REDUCE-IT and STRENGTH trials with omega-3 fatty acids: new analytical approaches

**DOI:** 10.3389/fnut.2024.1490953

**Published:** 2024-12-19

**Authors:** Weiguo Zhang, Dan Gan, Shaofeng Huo, Peng Chen

**Affiliations:** ^1^Las Colinas Institutes, Irving, TX, United States; ^2^R&D, Sirio Life Technology Co., Ltd, Shanghai, China; ^3^R&D, Sirio Pharma Co., Ltd, Shantou, Guangdong, China

**Keywords:** major adverse cardiovascular events (MACEs), residual cardiovascular risk (RCR), eicosapentaenoic acid (EPA), docosahexaenoic acid (DHA), resting heart rate (RHR), heart rate variability (HRV), cardiovascular aging, ethnic difference

## Abstract

Two large-scale, randomized, double-blind, placebo-controlled trials—REDUCE-IT and STRENGTH—have garnered significant attention in cardiovascular medicine. Both trials aimed to evaluate the effects of prolonged administration of nutritional lipids, specifically omega-3 fatty acids, on major adverse cardiovascular events (MACEs) in high-risk patients undergoing statin therapy. REDUCE-IT used eicosapentaenoic acid (EPA) ethyl ester with mineral oil as a control, while STRENGTH utilized a carboxylic acid formulation of both EPA and docosahexaenoic acid (DHA) with corn oil as a control. Notably, REDUCE-IT demonstrated a reduction in MACE risk with EPA, whereas STRENGTH showed no such benefit with the combination of EPA and DHA. Despite extensive and insightful discussions following the publication of these trials, the underlying reasons for this discrepancy remain elusive. We posit that further investigation into resting heart rate (RHR), heart rate variability (HRV), and ethnic subgroup data—collected but not fully explored—is critical to unraveling the divergent outcomes of the REDUCE-IT and STRENGTH trials. These additional analyses could provide pivotal insights into the mechanisms driving the differential effects of omega-3 fatty acids in high-risk cardiovascular patients. Given that previous discussions have not fully addressed these potential variables, exploring them may illuminate unexplored pathways and offer a deeper understanding of the mechanistic and clinical roles of omega-3 s in cardiovascular health. We hypothesize that by delving into these under-analyzed factors, we can not only clarify the discrepancies between the trials but also advance our broader understanding of cardiovascular nutrition and medicine.

## 1 Introduction

In recent years, clinical cardiovascular research has faced significant controversy regarding the chronic administration of nutritional lipids, specifically omega-3 fatty acids that belong to the family of long-chain polyunsaturated fatty acids, to reduce major adverse cardiovascular events (MACEs) in high-risk patients. Two major clinical studies, both multicenter, randomized, double-blind, placebo-controlled trials, have investigated this by administering omega-3 fatty acids, particularly eicosapentaenoic acid (EPA) and docosahexaenoic acid (DHA), alongside cholesterol-lowering statin therapy (1, 2). However, the results on MACE outcomes were conflicting: the Reduction of Cardiovascular Events with Icosapent Ethyl–Intervention Trial (REDUCE-IT) demonstrated that active intervention was effective. At the same time, the Statin Residual Risk with Epanova in High Cardiovascular Risk Patients with Hypertriglyceridemia trial (STRENGTH) concluded that such intervention was futile.

Omega-3 fatty acids are recognized for their broad, pleiotropic effects on human health, extending beyond cardiovascular protection (3).

Nonetheless, their primary clinical application remains the management of hypertriglyceridemia—a condition once overlooked as a traditional cardiovascular risk factor, but now recognized as a key contributor to residual cardiovascular risk (RCR) due to its association with vascular inflammation and atherosclerosis (4–7). Recent meta-analyses of 38 randomized controlled trials, involving 149,051 participants, suggest that both EPA monotherapy and combined EPA/DHA therapy reduce cardiovascular mortality and improve outcomes, with EPA showing a more pronounced benefit (8). Despite these promising findings, the conflicting results between the REDUCE-IT and STRENGTH trials highlight the need for deeper investigation into the mechanistic and clinical roles of omega-3 fatty acids in cardiovascular disease.

## 2 The trials with contrasting outcomes

The REDUCE-IT trial investigated the effects of a highly purified eicosapentaenoic acid (EPA) ethyl ester (icosapent ethyl), known commercially as VASCEPA, compared to a mineral oil placebo. This trial enrolled 8,179 patients with baseline plasma triglyceride levels ranging from 135 to 499 mg/dL (1.52 to 5.63 mmol/L) and low-density lipoprotein cholesterol (LDL-C) levels from 41 to 100 mg/dL (1.06 to 2.59 mmol/L). The primary efficacy measure was a composite of cardiovascular death, non-fatal myocardial infarction, non-fatal stroke, coronary revascularization, or unstable angina. The key secondary measure included cardiovascular death, non-fatal myocardial infarction, or non-fatal stroke. Over a median follow-up period of 4.9 years, the primary endpoint event occurred in 17.2% of patients in the icosapent ethyl group compared to 22.0% in the placebo group (hazard ratio 0.75; 95% confidence interval [CI] 0.68 to 0.83; *p* < 0.001). For the key secondary endpoint, the incidence was 11.2% in the treatment group compared to 14.8% in the placebo group (hazard ratio 0.74; 95% CI 0.65 to 0.83; *p* < 0.001). Additionally, the rate of cardiovascular death was 4.3% in the icosapent ethyl group compared to 5.2% in the placebo group (hazard ratio 0.80; 95% CI 0.66 to 0.98; *p* = 0.03) (1).

In contrast, the STRENGTH trial examined the effects of a carboxylic acid formulation of omega-3 fatty acids, combining EPA and docosahexaenoic acid (DHA), marketed as EPANOVA, against a corn oil placebo. This trial included 13,078 patients with a median plasma triglyceride level of 240 mg/dL (range: 192 to 309 mg/dL) and a median high-density lipoprotein cholesterol (HDL-C) level of 75 mg/dL (range: 56 to 99 mg/dL). The primary and secondary endpoints were comparable to those of the REDUCE-IT trial. However, an interim analysis revealed that the primary outcome occurred in 12.0% of patients receiving the omega-3 carboxylic acid compared to 12.2% in the control group (hazard ratio 0.99; 95% CI: 0.90–1.09; *p* = 0.84). The trial was prematurely terminated on the recommendation of the independent data monitoring committee due to a low probability of demonstrating any beneficial cardiovascular outcomes from the omega-3 carboxylic acid compared to corn oil. At study closure, patients had been followed for a median of 42.0 months, and the median treatment duration was 38.2 months (2).

The discrepancies between the REDUCE-IT and STRENGTH trials have sparked considerable debate in the field, particularly regarding effective strategies to reduce residual cardiovascular risk and mitigate MACE. Several theories have been proposed to explain these conflicting outcomes.

## 3 Explaining the contradictory results

Several explanations have been proposed by investigators and experts to account for the discrepancies observed between the REDUCE-IT and STRENGTH trials (9–14). Below are some of the dominant theories:

### 3.1 Differences in active oils

A significant factor contributing to the contrasting outcomes is the difference in omega-3 formulations used in the trials. REDUCE-IT used a high dose of purified EPA, while STRENGTH utilized a combination of EPA and DHA. In REDUCE-IT, the icosapent ethyl group received 3,840 mg of EPA daily, which significantly increased plasma EPA levels to 144.0 μg/mL at 12 months. In contrast, participants in STRENGTH received 2,200 mg of EPA and 800 mg of DHA daily, resulting in a lower plasma EPA concentration of 89.6 μg/mL.^1^ Research indicates that circulating omega-3 levels are inversely correlated with all-cause and cardiovascular mortality (15–17), with a certain threshold of circulating EPA required before clinical benefits manifest (18–20).

Structurally, EPA and DHA differ in their number of double bonds and hydrocarbon chain length: EPA has 5 double bonds and 20 carbons, while DHA has 6 double bonds and 22 carbons. These structural differences result in distinct biological effects when incorporated into cell membranes. EPA is more concentrated in the endothelial and other vascular cells, where it integrates into lipoprotein particles and cellular membranes in an extended conformation that helps preserve the membrane structure and normal distribution of cholesterol (21); longer hydrocarbon DHA, on the other hand, is more concentrated in the neuronal or neural tissues such as brain and eye retina and incorporated into the membrane structure that induces more isomerization and conformational changes, resulting in increasing membrane fluidity and promotion of cholesterol domains (21, 22).

Functionally, EPA is believed to have a greater capacity for scavenging reactive oxygen species (ROS) and promoting plaque stability by stabilizing unpaired electrons with its multiple conjugated double bonds (23), thereby reducing lipid oxidation and cholesterol crystal formation.

Beyond membrane mechanisms, EPA and DHA may differentially modulate signal transduction pathways and omega-3 receptors, influencing platelet aggregation and vascular dilation (24). For example, a study showed that purified EPA (commercially known as EPADEL) administered to patients on aspirin and clopidogrel therapy for 12 weeks following coronary stent implantation (25), likely mediated by resolvin E1 (RvE1), a pro-resolving mediator derived from EPA with anti-inflammatory and anti-thrombotic effects (26). Conspicuously, in REDUCE-IT, serious but non-fatal bleeding events occurred in 2.7% of patients who received VASCEPA, compared to 2.1% in the mineral oil group (1). In contrast, STRENGTH reported no excess bleeding events in the EPANOVA group compared to the corn oil control (2). Additionally, the STRENGTH trial reported that LDL cholesterol levels increased in the active treatment group but not in the corn oil group (1.2% vs. −1.1%; geometric mean ratio [GMR]: 1.03 [95% CI: 1.01–1.04]; *p* < 0.001), aligning with findings from other trials (27).

### 3.2 Difference in comparator oils

Another critical factor contributing to the contradictory results is the choice of control oils. REDUCE-IT used mineral oil as the comparator, while STRENGTH used corn oil. One prominent argument is that mineral oil may be pro-inflammatory and negatively impact lipid profiles. In REDUCE-IT, the levels of apolipoprotein B, LDL cholesterol, and hs-CRP increased in the mineral oil control group. For example, hs-CRP levels increased from a median value of 2.1 mg/L at baseline to 2.8 mg/L at year 2, marking a 33% increase. This deterioration in inflammation biomarkers and lipid profiles in the mineral oil group could partially explain the observed 25% reduction in MACEs in the icosapent ethyl group. Nonetheless, the FDA approved icosapent ethyl as an adjunctive therapy to reduce MACE risk, concluding that the effects of mineral oil were unlikely to fully account for the significant benefits observed (28).

Mineral oil, which is inert and minimally absorbed in the gastrointestinal tract, is often used as a mild laxative. However, concerns have been raised about its potential to interfere with the absorption of statins and fat-soluble vitamins (A, D, E, and K), possibly contributing to the negative changes in lipid profiles and hs-CRP observed in the REDUCE-IT control group. Previous studies investigating the relationship between mineral oil use and vitamin A absorption have yielded inconsistent results (29), and the literature on its effects on other fat-soluble vitamins remains limited.

Conversely, corn oil, used as a placebo in the STRENGTH trial, is rich in linoleic acid (an *ω*-6 polyunsaturated fatty acid), which may introduce a mild confounding effect. The dose of corn oil in STRENGTH accounted for approximately 1% of the study participants’ daily energy intake. Observational studies indicate that each 1% increase in polyunsaturated fatty acid energy is associated with a 3 to 7% reduction in cardiovascular events (11). Consequently, while corn oil may offer some mild benefits, identifying a truly nutritionally neutral placebo in trials of this nature can be challenging, if not impossible.

## 4 Ongoing efforts to resolve the puzzles

A straightforward question arises: can a new trial be designed to compare VASCEPA with an edible oil other than mineral oil? As of 25 October 2024, when this article was being drafted, 46 trials involving VASCEPA were registered on ClinicalTrials.gov.^2^ Among the cardiovascular trials, one active study titled *Efficacy of Ethyl Icosapentate in Patients with Severe Hypertriglyceridemia* (NCT04239950), commenced on 9 May 2020, with an enrollment of 300 participants and a completion date anticipated by July 2023. The specific control oil used in this trial may only be disclosed after the results are published. Another ongoing study, *Aggressive Risk-Prevention Therapies for Coronary Atherosclerotic Plaque [ARTCAP]* (NCT06280976), began on 1 March 2024, with 200 participants enrolled, and is projected to be completed by 31 January 2029. The control oil for this trial also remains undisclosed. However, regardless of the control oil utilized, these trials are not specifically designed to address the controversies surrounding the REDUCE-IT trial.

For EPANOVA, a similar inquiry may be less feasible. The trialists of the STRENGTH study argue that mineral oil contributes to adverse biochemical effects, particularly by unfavorably raising circulating CRP levels; and therefore should not be used as a comparator (2). As of 31 May 2024, when this article was being drafted, there were 18 trials registered with EPANOVA as an investigative drug on ClinicalTrials.gov,^3^ with no ongoing trials available. A recent update on 25 October 2024, confirmed no change in the total number of trials or trial activity status.

Given these circumstances, designing and executing new trials to address even some of the controversies stemming from REDUCE-IT and STRENGTH does not seem to be a priority in the cardiovascular research agenda. Several significant factors hinder further investigations, including financial constraints, limited funding, ethical considerations, and proprietary issues related to drug patents and intellectual property rights, which may restrict access to necessary compounds. Collectively, these challenges complicate efforts to decipher the complexities involved.

## 5 New analytical proposals

As mentioned earlier, it is unlikely that the gaps between the REDUCE-IT and STRENGTH trials will be closed anytime soon. In the meantime, several articles published in recent years offer insightful and valuable perspectives through editorials, commentaries, and reviews (10–14). While the primary medical use of EPA and DHA is to manage elevated triglyceride levels in the bloodstream, scientific evidence also highlights the pleiotropic functions of omega-3 fatty acids on the cardiovascular system (9). Consequently, the following new analytical strategies are proposed to examine existing datasets, which may help uncover the underlying reasons for the differing results of the two trials.

### 5.1 Resting heart rate (RHR)

Clinical and epidemiological studies have shown that individuals with higher RHR are at an increased risk of morbidity and mortality (30–33). Elevated RHR is now recognized as an emerging risk factor for cardiovascular morbidity and mortality (30, 33, 34). In homeothermic mammals, the total number of heartbeats over a lifespan appears to be finite; those with faster heart rates may exhaust their heartbeat allotment more quickly, making them more susceptible to age-related disorders and likely to have shorter lifespans (30). Supplementation with omega-3 fatty acids has been found to exert a negative chronotropic effect on heart rate (35). A recent meta-analysis involving approximately 3,000 participants from 51 randomized controlled trials (RCTs) reported that omega-3 fatty acids significantly reduced heart rate by a mild but notable −2.23 beats per minute (bpm; 95% CI: −3.07, −1.40 bpm) (36). The mechanisms by which omega-3 fatty acids modulate heart rate are 2-fold: (1) they influence cardiac automaticity by regulating ion movement through potassium, sodium, and calcium channels within the cardiomyocytes of the sinoatrial node, the heart’s natural pacemaker. This modulation affects the spontaneous depolarization and repolarization cycle of pacemaker cells, thereby impacting the heart’s rhythmic contractions (35); and (2) they modulate the autonomic nervous system, balancing sympathetic and parasympathetic outputs in response to internal and external stimuli. This modulation primarily takes place in the brain’s autonomic centers, enhancing parasympathetic activity and increasing the release of neurotransmitters, such as acetylcholine, at peripheral efferent nerve terminals (35). For more detailed insights on the modulation of potassium, sodium, and calcium channels by omega-3 fatty acids, as well as their central effects at the brainstem level, please refer to reference (35).

RHR was assessed at baseline and during follow-up observations in both the REDUCE-IT and STRENGTH trials, indicating that these data should be accessible for analysis. Investigating heart rate responses to both active and placebo treatments over time is highly encouraged. Understanding whether heart rate reduction contributed to clinical endpoints, in addition to existing analyses and assumptions, is crucial. Given the high concentrations of EPA and EPA + DHA used, along with the substantial populations studied—4,089 participants in REDUCE-IT and 6,539 in STRENGTH, compared to only 1,656 participants who received omega-3 in the previous meta-analysis (36)—this proposed analysis could provide valuable insights into the contrasting outcomes of the two trials and underscore the effects of omega-3 fatty acids on heart rate.

Previously, the investigators of the Systolic Hypertension in Europe (Syst-Eur) Trial analyzed the role of heart rate in predicting mortality within the control arm, which included 2,293 men and women who received a placebo. This study focused on elderly patients with hypertension, assessing the impact of heart rate on both all-cause and cardiovascular mortality. Over a follow-up period of 4 years, individuals with heart rates exceeding 79 bpm exhibited a 1.89 times higher risk of all-cause, cardiovascular, and non-cardiovascular mortality than those with heart rates at or below 79 bpm. Additionally, in a subgroup of 807 subjects who underwent ambulatory monitoring, both clinic and ambulatory heart rates were found to predict non-cardiovascular mortality, with clinic heart rate showing a significant association in the Cox regression analysis (37).

Analyzing heart rate data from the REDUCE-IT and STRENGTH trials using a methodology similar to that of the Syst-Eur Trial could provide new insights into the relationship between heart rate and mortality, particularly regarding cardiovascular events. Previous studies have supported a hypothesis regarding a positive relationship between resting heart rate and the pathogenesis of cardiovascular disease (38–41). It is proposed that individuals with higher baseline heart rates, who maintain elevated heart rates in either the control or treatment groups during the study, or those who exhibit less responsiveness to the bradycardic (i.e., negative chronotropic) effect of omega-3, may experience higher rates of cardiovascular events, including mortality.

### 5.2 Heart rate variability (HRV)

Heart rate variability (HRV) refers to the natural fluctuations in the time intervals between successive heartbeats, typically measured using an electrocardiogram (ECG) or 24-h Holter monitor or peripheral photoplethysmography. Unlike a metronome, where each beat is perfectly timed, the heart’s rhythm varies even when the ECG shows a normal sinus rhythm (42). HRV reflects the complex interplay between the heart’s intrinsic automaticity and the autonomic nervous system, which consists of both sympathetic and parasympathetic branches. These systems continuously adjust the heart’s activity in response to internal and external stimuli, modulating beat-to-beat changes to maintain balance (43). Given that altered intercellular communication is a hallmark of aging (44), this variability sheds light on the adaptability and efficiency of brain–heart communication, which encompasses afferent sensing, signal relay and processing, efferent innervation, and subsequent cardiac response. In such regards, HRV serves as a valuable marker of autonomic function, cardiac health, psychological states, nutrition status, and overall wellbeing (45, 46), as well as aging status (47–49).

Population studies have shown that higher HRV is generally observed in young, healthy individuals, while a decline in HRV is associated with the aging progress (50–52). Reduced HRV, or specific HRV indices, have been linked to increased cardiovascular mortality and arrhythmic complications, especially in post-myocardial infarction patients and in the general population (52–55). On the other hand, higher HRV, as reported, is associated with increased longevity (56, 57) and a reduced risk of morbidity.

Omega-3 fatty acid supplementation has been shown to improve both time-domain and frequency-domain indices of HRV (43, 58–61). While not all HRV measures may correlate linearly with age, HRV remains a recognized surrogate marker for aging and a predictor of premature mortality. Although the STRENGTH trial did not record ECG data post-randomization,^4^ the REDUCE-IT trial did collect standard 12-lead ECGs at baseline and annually thereafter according to the Study Protocol and Statistical Analysis Plan.^5^ Analyzing the effect of VASCEPA on HRV in both treatment and control groups offers valuable insights into how long-term omega-3 supplementation may enhance HRV, potentially contributing to reduced major adverse cardiovascular events (MACEs) and improved survival rates.

### 5.3 Ethnic subgroup analysis

The results of the STRENGTH trial were not entirely null. A prespecified analysis within the Asian subgroup, representing 10% of the total study population, showed a notable benefit from omega-3 treatment. The primary composite cardiovascular endpoint rate was 3.63 per 100 person-years in subjects who received active omega-3 treatment, compared to 5.08 per 100 person-years in those who received corn oil–even though the trial was prematurely terminated (2). The difference was 1.45%, and the hazard ratio was 0.72 (95% CI: 0.54–0.96), which is comparable to the overall hazard ratio reduction of 0.75 (95% CI: 0.68–0.83) reported in the REDUCE-IT trial (2). Additionally, the hazard ratio in the Asia region was 0.78 (95% CI: 0.62–0.98). The ethnic differences observed in the STRENGTH study warrant further investigation to determine whether these discrepancies are rooted in genetic factors, differences in chronic inflammation biomarkers and lipid profiles, dietary preferences, variations in disease development patterns (such as cerebrovascular or stroke vs. cardiac events), or other underlying mechanisms (such as hemodynamics or blood pressure). In REDUCE-IT, while patients in the Asia-Pacific region exhibited greater risk reductions for both primary and secondary composite endpoints with VASCEPA treatment, their sample size was limited—130 patients in the VASCEPA group and 132 in the control group, or 3.2% of the overall study population—yielding results that, though promising, lacked statistical significance (1). Importantly, variations in response to omega-3 supplementation across ethnic groups have also been observed in other studies, though these require further validation and mechanistic insight (62, 63). To reveal these details not only helps explain why the results are inconsistent between ethnic groups within the STRENGTH trial but also clarifies the context in which EPA + DHA can work without having mineral oil as a placebo control, which, as discussed before, was suspected to contribute to the observed reduction in MACEs.

### 5.4 The importance of these new analyses

The analytical strategies outlined above are not just academic exercises; they hold the potential to illuminate critical aspects of omega-3’s effects that have been previously overlooked or underappreciated. By revisiting and analyzing existing datasets with a fresh perspective—whether through heart rate modulation, heart rate variability, or ethnic subgroup differences—researchers can gain a deeper understanding of the pleiotropic effects of omega-3 fatty acids. These efforts could not only clarify the seemingly contradictory results between REDUCE-IT and STRENGTH but also inform more precise therapeutic approaches tailored to different populations. In doing so, these analyses may significantly impact clinical decision-making and future therapeutic recommendations, ultimately improving patient outcomes in cardiovascular health.

## 6 Conclusion and perspective

Reanalyzing the existing datasets from the REDUCE-IT and STRENGTH trials presents a valuable opportunity to uncover common denominators that could reconcile their divergent outcomes, or at least shed light on the mechanisms driving their discrepancies. Specifically, focusing on the effects of resting heart rate, heart rate variability, and ethnic subgroup differences in response to EPA or the combination of EPA and DHA could provide novel insights or significantly reinforce the current evidence base.

Much of the research conducted thus far has been grounded in nutritional surveys or short-term dietary interventions, often with lower omega-3 dosages that may not capture the full spectrum of effects observed in large-scale clinical trials. Delving into these deeper analyses, whether undertaken by the original trial investigators or by independent experts with access to the data, could yield pivotal findings that inform future clinical guidelines.

These investigations hold the potential to refine both dietary and pharmaceutical strategies for reducing major adverse cardiovascular events and addressing residual cardiovascular risk, ultimately advancing cardiovascular health and longevity. By expanding our understanding of omega-3’s pleiotropic effects through these new analytical perspectives, we can further enhance evidence-based approaches for cardiovascular disease prevention, treatment, and sustained health.

## Footnotes

^1^https://www.acc.org/education-and-meetings/image-and-slide-gallery/media-detail?id=062fc9e4b3a74a9fb1c196a35dad8f3b, accessed on 30 July 2023.

^2^ https://clinicaltrials.gov/search?intr=Vascepa

^3^ https://clinicaltrials.gov/search?term=EPANOVA

^4^https://storage.googleapis.com/ctgov2-large-docs/17/NCT02104817/Prot_000.pdf under https://clinicaltrials.gov/study/NCT02104817, accessed on 30 December 2023.

^5^https://storage.googleapis.com/ctgov2-large-docs/61/NCT01492361/Prot_SAP_000.pdf under https://clinicaltrials.gov/search?intr=Vascepa&term=NCT01492361, accessed on 30 December 2023.
